# Memory First Aid: remote memory service and webinar-based dementia training for non-medical graduates in Nepal, India, Pakistan and Sri Lanka

**DOI:** 10.1192/bji.2020.42

**Published:** 2020-07-30

**Authors:** Arun Jha, Shehan Williams, Bhaweshwar Singh, Prabhat Pradhan, Khem Raj Bhatt, Muhammad Iqbal Afridi, Rahul Tomar, Kaushik Mukhopadhaya

**Affiliations:** 1 Consultant Old Age Psychiatrist, Hertfordshire Partnership University NHS Foundation Trust, St Albans, UK. Email: arunjhauk@gmail.com; 2 Professor in Psychiatry, Faculty of Medicine, University of Kelaniya, Sri Lanka; 3 Professor of Zoology, Institute of Gerontology and Geriatrics, LN Mithila University, Darbhanga, India; 4 Executive Member, Alzheimer's and Related Dementias Society, Kathmandu, Nepal; 5 Assistant Professor, Central Department of Psychology, Tribhuvan University, Kathmandu, Nepal; 6 Professor and Head, Department of Psychiatry and Behavioural Sciences, Jinnah Postgraduate Medical Centre, Karachi, Pakistan

**Keywords:** Dementia, first aid, remote, South Asia, Nepal

## Abstract

The prevalence of dementia is rising in low-resource countries, where specialist memory services are almost non-existent. The COVID-19 pandemic has created opportunities for innovative remote healthcare. Research shows a lack of dementia literacy and help-seeking behaviour for memory-related problems among older adults in South Asian countries. This paper proposes a remote memory service model and virtual dementia training in South Asian countries, called Memory First Aid (MFA). MFA offers help to a person experiencing memory difficulties until appropriate professional help is received. The MFA course is a 12-h webinar-based package consisting of four weekly modules. It covers dementia awareness and clinical features. The aim is to develop a non-medical workforce able to screen and assess older people with suspected dementia.

Dementia is a rapidly growing public health problem affecting around 50 million people worldwide, with approximately 60% living in low- and middle-income countries (LMICs). This figure is set to triple by 2050. International reports indicate a growing number of people with dementia in South Asian countries^1,2^ (Table 1).

**Table 1:** Projected population and number of people with dementia in South Asia in 2015–2050

	Population (thousands), 2015	Estimated number of people with dementia (thousands)		
		2015	2030	2050
India	1 282 390	4031	6743	12 542
Nepal	28 441	78	134	285
Sri Lanka	21 612	147	262	463
Pakistan	188 144	450	712	1422
Bangladesh	160 411	460	834	2193
Total Asia Pacific	3 991 793	23 279	39 409	70 981

Source: Alzheimer's Disease International & Alzheimer's Australia.^1^

Many South Asians view memory loss as a normal part of ageing or understand symptoms of dementia through religious belief.^3^ Providing affordable and sustainable dementia care services in these countries poses numerous challenges. COVID-19 has forced rapid changes in global healthcare, with a significant increase in remote consultations to enable people to access healthcare during physical distancing. Remote healthcare poses specific challenges for memory services owing to patients’ cognitive impairment and the reliance of the clinician on relatives. In May 2020, NHS England's London Clinical Network distributed its *Guidance on Remote Working for Memory Services during COVID-19* to staff (this is not on the LCN website but copies may be found online). At the same time, we planned an innovative webinar-based dementia course called Memory First Aid (MFA). MFA aspires to train a pool of non-medical graduates in South Asian countries to offer dementia screening and brief assessment. The course is adapted from mental health first aid courses run in Australia,^4^ Nepal^5^ and elsewhere. We have also planned a post-COVID-19 remote memory service based on the Rural and Remote Memory Clinic (RRMC) project, which reported high patient and caregiver satisfaction with telehealth video conferencing.

## The Memory First Aid pathway and action plan

**Fig. 1:**
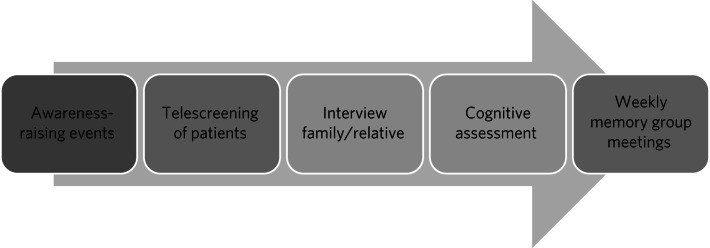
Components of the Memory First Aid pathway.

Figure 1 depicts the MFA pathway, which consists of screening, assessment and post-diagnostic support. On this pathway, the local branch of Memory First Aid International will organise awareness-raising events along with the nearest Alzheimer's Society and/or similar organisations. The local MFA centre will have a helpline to offer a free memory screening service. All individuals who screen positive will be offered a brief initial assessment using the Rowland Universal Dementia Assessment Scale (RUDAS) cognitive test.

RUDAS is a copyright-free instrument particularly useful for people in low- and middle-income countries where literacy or education is low.^6^ RUDAS is a short interview-based questionnaire that assesses multiple cognitive domains, including memory recall, visuospatial orientation, praxis, visuoconstructional drawing, judgement and language. It has been validated in Nepal (Nepali-RUDAS)^7^ and is relevant to all South Asian countries.

**Fig. 2:**
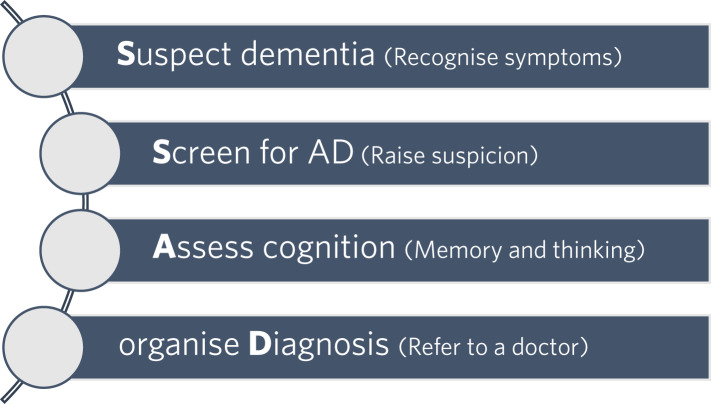
The Memory First Aid action plan. AD, Alzheimer's disease.

In any first aid course, participants learn an action plan for the best way to help someone who is injured or ill. For example, in the UK, when ambulance paramedics are trained to recognise the symptoms of stroke, they are taught to remember the mnemonic FAST, which stands for: Face (can the person smile?), Arms (can the person raise both arms?), Speech problems (can the person speak clearly and understand what you say?) and Time (If you see any of these three signs, it's time to call 999). The MFA course provides an action plan on how to help a person experiencing memory difficulties. Its mnemonic is SSAD: Suspect dementia, Screen for Alzheimer's disease, Assess cognition and organise Diagnosis (Fig. 2).

## Course content

The MFA course teaches volunteers how to recognise the symptoms and signs of Alzheimer's dementia, how to screen older people with memory problems for dementia, how to offer basic cognitive assessment, and how to organise diagnostic assessment for people with suspected dementia.

MFA is a 12-hour webinar-based course consisting of four modules (3 h each) delivered over 4–6 weeks. The course is based on tier 1 and tier 2 of the Dementia Training Standards Framework developed by NHS Health Education England in 2018.^8^ Tier 1 is related to dementia awareness raising, in terms of knowledge, skills and attitudes for all those working in health and care settings. Tier 2 is about knowledge, skills and attitudes for roles that have regular contact with people living with dementia. Table 2 lists the key subject areas and learning outcomes for the four MFA modules.

**Table 2:** Key subject areas and learning outcomes for the modules of the Memory First Aid course

Module	Subject area	Key learning outcomes
1	Dementia awareness	The learner will: know the meaning of the term dementia and the importance of family caregivers be aware of the prevalence of dementia in South Asia be able to recognise signs of various types of dementia, particularly vascular dementia and Alzheimer's disease know what actions individuals can take to reduce the risk of dementia or to delay onset know why early diagnosis of dementia is important be aware of the impact of dementia on individuals, families and society be able to communicate effectively and compassionately with individuals who have dementia understand reasons why a person with dementia may exhibit signs of distress and how behaviours seen in people with dementia may be a way of communicating unmet needs
2	Dementia identification, assessment and diagnosis	The learner will: be sensitive to people's vision, hearing, language and literacy problems and to sociocultural norms know the most common types of dementia and their causes know why early diagnosis of dementia is important and the likely outcomes if assessment and treatment are delayed know the progressive nature of dementia and some of the major impairments and difficulties people may face as dementia progresses be able to appropriately refer patients to access nearest specialist services and support networks
3	Pharmacological interventions in dementia care	The learner will: know the most common medications used to treat the symptoms of dementia know the main risks and benefits of using antipsychotics, antidepressants, anxiolytics, anticonvulsants and cognitive enhancers and be aware of the impact drugs may have on daily living, including common side-effects such as taste disturbances and a dry mouth
4	Health and well-being in dementia care	The learner will: be able to communicate the importance of exercise for the patient and caregiver as well as to prevent dementia understand the importance for individuals with dementia to maintain good physical, mental and oral health through food, drink, exercise and a healthy lifestyle that includes social engagement understand triggers and responses to stressed and distressed behaviours understand the role of family and carers in supporting the health and well-being of people with dementia be aware of the benefits and limitations of medication to manage behavioural and psychological problems, including associated risks

## Pilot testing

Evaluation of the course at three pilot sites – Kathmandu in Nepal, Darbhanga in India and Colombo in Sri Lanka – will commence in September 2020. Its effectiveness will be measured using the pre- and post-test Alzheimer's Disease Knowledge Scale (ADKS).^9^ This method has been previously used by authors in the UK for a similar course, Dementia First Aid, for family caregivers of people with early dementia.^10^ The success of the programme will be measured by the number of people with suspected dementia being screened and diagnosed. Patient and caregiver satisfaction with telescreening will be evaluated using the Telehealth Satisfaction Scale (TeSS).^11^ If successful, the MFA course and tele-memory service will be rolled out to other South Asian countries.
